# Mouse models of glioblastoma for the evaluation of novel therapeutic strategies

**DOI:** 10.1093/noajnl/vdab100

**Published:** 2021-07-26

**Authors:** Alexander F Haddad, Jacob S Young, Dominic Amara, Mitchel S Berger, David R Raleigh, Manish K Aghi, Nicholas A Butowski

**Affiliations:** 1 Department of Neurological Surgery, University of California, San Francisco, California, USA; 2 Department of Radiation Oncology, University of California, San Francisco, San Francisco, California, USA

**Keywords:** murine models, glioblastoma, tumor, U87, GL261

## Abstract

Glioblastoma (GBM) is an incurable brain tumor with a median survival of approximately 15 months despite an aggressive standard of care that includes surgery, chemotherapy, and ionizing radiation. Mouse models have advanced our understanding of GBM biology and the development of novel therapeutic strategies for GBM patients. However, model selection is crucial when testing developmental therapeutics, and each mouse model of GBM has unique advantages and disadvantages that can influence the validity and translatability of experimental results. To shed light on this process, we discuss the strengths and limitations of 3 types of mouse GBM models in this review: syngeneic models, genetically engineered mouse models, and xenograft models, including traditional xenograft cell lines and patient-derived xenograft models.

Preclinical models of cancer, including glioblastoma isocitrate dehydrogenase (IDH) wild-type (GBM), are essential to understand tumor biology and treatment.^1^ While a variety of animal models are used to study glioblastoma, the overwhelming majority of preclinical investigations involve mice.^2–4^ Mouse models are typically grouped into three categories: syngeneic models, genetically engineered mouse models (GEMMs), and xenograft models, including cell line-based xenografts and patient-derived xenografts (PDX), each of which has distinct advantages and disadvantages for modeling GBM biology and testing developmental therapeutics.^5^ The ideal model recapitulates key characteristics of human GBM that impact survival and response to treatment, including, but not limited to, the histological features of invasiveness, intra-tumoral genetic heterogeneity, tumor metabolism, the immune microenvironment^6^ (e.g., low immunogenicity of the tumor with low tumor mutational load and MHC I expression^7^)^8,9^ and genetic profile. Model response to existing treatments, such as radiotherapy and temozolomide, should also be similar to GBM (i.e., models should display appropriate chemo and radioresistance). Though no model exactly replicates human glioblastoma, each has unique features that should be considered when designing experiments or interpreting preclinical data. Moreover, the high number of successful preclinical treatments that have failed in subsequent human clinical trials highlights the imperfections of the available models and the importance of appropriate preclinical model selection.^10^ In this review, we provide an overview of the conventional murine models of GBM and their unique characteristics (Figure 1). We then discuss what preclinical treatment studies these models are suited for and the advantages/disadvantages of each model, as well as future directions in preclinical GBM modeling.

**Figure 1.**
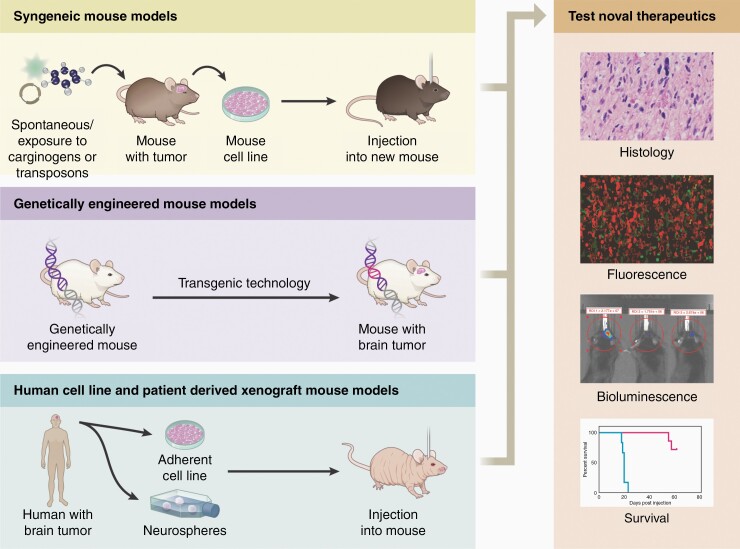
Schematic of the various mouse models available to glioblastoma researchers including syngeneic, genetically engineered, and xenograft models.

## Syngeneic Murine Models of GBM

### Introduction to Syngeneic Murine Models of GBM

Syngeneic models involve glioblastoma tumor cells that are murine in origin and can be transplanted back into mice of a similar genetic background. Syngeneic tumor lines can be generated from spontaneously occurring murine tumors,^11^ but are frequently generated using mutagenic chemicals^3,11^ or transposons.^12^ Syngeneic models are well suited for preclinical studies evaluating treatments involving the immune system, such as checkpoint inhibitors,^12^ as the immune system is intact in these models.

### Advantages and Disadvantages

Syngeneic models have multiple advantages, including that tumor cells can be easily maintained and expanded in vitro before their implantation into a mouse, resulting in the formation of consistent, reproducible tumors with reliable rates of growth and impact on murine survival. (Table 1)The ability to be implanted into immunocompetent animals provides a complete tumor microenvironment, as the host immune system is intact and able to be interrogated. Indeed, syngeneic cells are preferred over other model types, such as xenograft lines, in studies where a fully functional immune system is required, such as preclinical investigations into the use of immunotherapies.

**Table 1.** Summary of GBM Model Advantages and Disadvantages

Mouse Model	Originating Species	Uses in preclinical studies	Pros	Cons	Comments
GL261	Mouse	- Gene therapy, immune cell transfer, monoclonal antibodies, and cytokine therapies.^13,14^ - Checkpoint inhibitors.^15^ - Dendritic cell vaccines.^16,17^ - Investigate GSCs.^14,18^	- Can be used in immunocompetent mice. - Widely used and available.	- Highly immunogenic relative to human GBM - Underlying genetics are different than human GBM.^19^ - Homogeneous population of cells. - Significant variability between studies.	
SMA-560	Mouse	- Cytokine therapies.^20^ - Soluble CD70.^21^ - CAR T cells.^22^	- Can be used in immunocompetent mice. - Resistant to TMZ. - Secretes TGF-B, can be used to study immunosuppression in GBM.^23^ - Spontaneous tumor, not chemically induced.	- Immunogenic relative to human GBM. - Less commonly used. - Not as well characterized as other models. - Homogeneous population of cells.	
CT-2A	Mouse	- Evaluation of GSCs.^24^ - Immunotoxin against EGFRvIII.^25^	- Can be used in immunocompetent mice. - Can be used to study GSCs.	- Tumor mutational burden not well characterized. - Likely more immunogenic than human GBM. - Moderately utilized in the literature - Low invasion into surrounding brain parenchyma.^26^ - Homogeneous population of cells	- Increased immune suppression and aggressiveness when grown as neurospheres.^24^
SB28	Mouse	- Checkpoint inhibitors.^12^ - Anti-CD40 treatment.^27^	- Can be used in immunocompetent mice. - Poorly immunogenic, most closely represents tumor microenvironment of human GBM amongst syngenic models.	- Used in less than 15 publications to date, requires additional histologic and microenvironment characterization. - Homogeneous population of cells	
U251	Human	- Alkylating agents: temozolomide, lomustine, and carmustine as well as the anti-angiogenic small molecule drug cilengitide.^28^ - Bevacizumab.^28^ - Synergism between metformin and temozolomide.^29^	- Extensively used in over 1000 studies.^30,31^ - Histologically recapitulates GBM well.^32–35^	- Questions regarding authenticity and inter-lab variability.^32,36^ - Requires the use of immunodeficient mice. - Sensitive to TMZ and XRT- does not recapitulate human GBM response to treatment. - Lack intra-tumoral heterogeneity.	
U87	Human	- Anti-angiogenic therapies.^37^ - Siroliumus and chloroquine in combination with temozolamide.^38^ - Neural stem cells carrying tumoricidal gene products.^39^	- Used in over 2000 studies.^30^ - Genetically similar to human GBM—carries hTERT, PTEN, and ATRX mutations.^40,41^	- Histologically, tumors are not invasive.^32,33,42^ - Lack other histologic characteristics of human GBM as well.^32,33,35,42^ - Questions regarding authenticity, inter-lab variability.^43^ - Requires immunodeficient mouse. - Lack intra-tumoral heterogeneity.	
GEMMs	Mouse	- Used in a variety of studies investigating treatments and underlying molecular pathways of GBM.^42,44,45^ - TMZ treatment response.^46^ - Response to Hsp90 inbibitor.^47^ - Response to PARP inhibitor.^48^	- Can directly investigate impact of underlying tumor genetics on treatment response.^46–50^ - Does not require intracranial injection. - Develop more similarly to human GBM.^30^	- Lack intra-tumoral heterogeneity.^28,30,44^ - Can require sophisticated breeding strategies. - Can be slow to form or inconsistent. - Can be expensive - Tumor formation is variable, limiting the use of precise treatment delivery modalities.	
PDX	Human	- GSCs and response to treatment.^51,52^ - Pharmaceuticals: bevacizumab, TMZ, veliparib.^53–55^ - Oncolytic herpes virus. - High-throughput drug screens.	- Best recapitulates human GBM histology and heterogeneity.^51,56^ - Intra-tumoral heterogeneity.	- Variability between lines. - Usually requires immunodeficient mouse. - Can be difficult to establish, requires significant expertise.^57,58^	

However, there are disadvantages associated with syngeneic models as well. Of course, with any model that involves a cell line that is cultured in vitro in the presence of serum, these cells are susceptible to genetic drift and changes to the tumor cells over time.^30,36,59^ Also, while syngeneic models do allow for an intact immune system, that does not ensure that each cell line models the actual tumor microenvironment seen in human GBM; some syngeneic models provide more accurate tumor microenvironments than others.^12^ Moreover, human GBM cells have a relatively low mutational burden compared to other aggressive malignancies,^60^ which may limit the efficacy of immunotherapy.^61^ In contrast, many of the syngeneic murine GBM models carry high mutational burdens frequently hundreds of folds higher than human GBM.^12,62^ This is especially true for models that are chemically induced. The differences in mutational burden and immune microenvironments between popular syngeneic models and human GBM may contribute to the disconnect seen between the success of preclinical immunotherapies and the failures of subsequent human clinical trials. Chemically induced models are also often mismatch repair (MMR) deficit which may recapitulate hypermutated GBM, but can reduce the sensitivity of cells to alkylating agents, such as temozolomide.^63^ However, there are newer syngeneic models that better recapitulate the mutational load and microenvironment of human GBM, like the SB28 model, which was generated via oncogenic transposons rather than chemicals.^12^

Several syngeneic mouse models exist, such as SMA-560, CT-2A, GL261, and SB28 models. CT-2A and GL261 models have been generated using chemical induction methods, with GL261 being one of the most frequently used tumor models. Below, we expand on some of the most commonly used syngeneic cell lines.

### Brief Description of Syngeneic Models

#### ***GL261***.

—The GL261 line was created in 1970 via chemical induction with methylcholanthrene pellets implanted into the brains of C57BL/6 mice, then maintained by direct transfer of tumors.^3,13^ Stable cell lines were cultured in the 1990s. GL261 tumors resemble ependymoblastomas on histology, but otherwise recapitulate GBM phenotypes.

#### ***Pros***.

—The benefits of the GL261 model relate to its broad use and extensive characterization throughout the literature. As previously mentioned, GL261 models recapitulate GBM histology well. In addition, GL261 cells are easily maintained in culture, allowing them to be investigated in vitro and easily expanded for in vivo tumor injections. When grown in serum-free media, GL261 cells express CD133, a stem cell marker, and have increased tumorgenicity^18^ and immunogenicity.^14^ Thus, GL261 cells can be used to investigate glioma stem cells, which contribute to chemo- and radioresistance in human GBM.

With regards to conventional treatments, such as chemo- and radiotherapy, GL261 moderately recapitulates the response of human GBM. Similar to human GBM, local radiation therapy in GL261 tumors slows tumor growth without a long-term survival benefit.^19^ GL261 is also resistant to temozolomide (TMZ) in cell culture.^64^ TMZ treatment has also been shown to prolong the survival of mice with GL261 tumors, although, similar to radiotherapy, it does not lead to long-term survival.^65^ In addition to moderately recapitulating the human GBM response to conventional treatments, one of the largest benefits of the GL261 model is the ability for tumor cells to be implanted into immunocompetent C57/BL6 mice without rejection, due to their C57/BL6 background. As a result, the majority of experiments utilizing GL261 are those investigating immunotherapies or other treatments reliant on the immune system.

#### ***Cons***.

—While frequently used to evaluate immunotherapeutics, GL261 tumor models may not accurately recapitulate the immunogenicity of human GBM. GL261 is moderately immunogenic relative to human GBM. In fact, unlike human GBM, GL261 has high MHC I expression and a high tumor mutational load, with over 4932 non-synonymous exome mutations and a high number of predicted neoepitopes.^62^ As a result, 90% of mice prevaccinated with irradiated GL261 cells fail to form a tumor.^19^ This immunogenicity may explain the number of successful preclinical immunotherapy studies in GL261, including immune checkpoint blockade, but the subsequent failure of these treatments in clinical trials.^12,15,66^ It is also worth noting that the characteristics of GL261 tumor cells, like many other cell types, likely change following the unique pressures of long-term cell culture, making it possible that GL261 cell lines differ between labs. This variability is highlighted by the large range (10^4^–10^6^) of implanted GL261 cells needed to observe tumor formation in the literature, although this may also be in an effort to increase or shorten survival curves.^12,13,67^ Genetically, GL261 cells have been reported to have elevated p53 expression and carry a p53 point mutation; this is also seen in many human GBMs and is associated with a worse prognosis.^19,68^ However, GL261 also carries a mutation in the K-Ras oncogene and has elevated c-myc expression, which are not typically seen in human GBM.^19^ While GL261 cells carry wild-type cytosolic isocitrate dehydrogenase (IDH1), the introduction of the R132H IDH mutation has been shown to increase survival following immunization with a peptide vaccine encompassing the mutation site, further highlighting the model’s immunogenicity.^69^

#### ***Uses***.

—The GL261 mouse model has been used extensively in the literature and is currently the most common murine GBM model, especially as it is reproducible and easy to use. GL261 has been used to study a variety of topics within GBM research, including gene therapy, immune cell transfer, monoclonal antibodies, and cytokine therapies.^13,14^ However, unlike human GBM, the GL261 model has been shown to respond well to checkpoint inhibitors against PD-L1, CTLA-4, and IDO.^15,70–72^ Indeed, in a study by Wainwright et al., 100% of mice with GL261 tumors treated with combination therapy against PD-L1, CTLA-4, and IDO demonstrated long-term survival with reductions in tumor-infiltrating regulatory T cells (Tregs).^15^ Given the high neoantigen load of GL261 and the unique tumor antigens it expresses, including EphA-2, GARC-1, and HMP/AN2, dendritic cell vaccines have also been successful in treating mice with GL261 tumors.^16,17^

#### ***CT-2A***.

—Similarly to the GL261 model, CT-2A was chemically induced with methylcholanthrene in C57BL/6 mice.^73^ The CT-2A model accurately recapitulates several characteristics of high-grade gliomas, including high cell density, elevated mitotic index, nuclear polymorphisms, hemorrhage, microvascular proliferation, and pseudopalisading necrosis.^74^

#### ***Pros***.

—While less commonly used than GL261, the CT-2A model has some distinct benefits. As previously mentioned, CT-2A tumors recapitulate human GBM histologically and display some of the key characteristics associated with human GBM, such as pseudopalisading necrosis.^74^ Genetically, CT-2A tumors are p53 wild-type and PTEN deficient, also similar to some human GBMs.^74–76^ Of note, CT-2A cells can also be used to model glioma stem cells; the invasiveness and proliferation of CT-2A cells can be significantly increased when brain tumor stem cell growth conditions are provided.^75^ This is a key characteristic of the CT-2A model that has been utilized in previous studies and is a growing area of use for the model.^24^ An additional advantage of CT-2A cells is their high tumorigenicity; mice have a median survival of 20 days following an intracranial injection of 1x10^4^ cells, making them amenable to in vivo experiments with shorter durations.^77^

#### ***Cons***.

—While they are highly tumorigenic and proliferative, CT-2A tumors tend to have low invasion into surrounding brain parenchyma with clearly defined borders, unlike human GBM.^26^ The immunogenicity of CT-2A is also higher than human GBM. Although there is a paucity of data on the tumor mutational burden of CT-2A, it is likely high as a result of the chemical induction method used to generate the cell line. In addition, MHC I is expressed by CT-2A cells and is upregulated in response to interferon-gamma, further contributing to the immunogenicity of the model.^78^ An additional disadvantage of the CT-2A model is its relatively poor characterization. For example, although frequently described as chemo and radioresistant,^74,79^ a paucity of data exists on the specific effects of TMZ and radiotherapy on tumor growth in CT-2A, likely due to its use mainly for the investigation of immunotherapeutics.

#### ***Uses***.

—Recent studies have highlighted the ability of neurosphere CT-2A cells to facilitate immune suppression in addition to displaying a more aggressive phenotype relative to CT-2A cells grown in a monolayer.^24^Though less popular than other models, such as GL261, CT-2A has also been used to study a wide range of other potential GBM treatments. Ladomersky et al. demonstrated that triple therapy of whole-brain radiotherapy, immune checkpoint blockade (PD1 mAb), and BGB-5777 (an IDO inhibitor) significantly increased the survival of mice with intracranial CT-2A tumors, with 25% demonstrating a long-term survival benefit.^80^ A CT-2A-EGFRvIII model has also been used to highlight the efficacy of an immunotoxin against epidermal growth factor receptor (EGFR) and EGFRvIII with a significant survival benefit in treated mice relative to controls.^25^ Thus, CT-2A is a versatile model that, although in need of further characterization, will likely be used in a number of future studies, especially those involving novel immunotherapeutics.

#### ***SMA-560***.

—The SMA-560 model is another frequently used syngeneic model. It is spontaneously derived, rather than chemically induced, and not quite as well characterized as the GL261 model. The cell line stems from a spontaneous glioma in the VM mouse strain, initially described in 1971, with tumorigenic cell lines established in 1980.^11,81^ Thus, the model is used in VM/Dk mice. SMA-560 tumors resemble anaplastic astrocytoma, as it displays infrequent necrosis histologically. It also seems to recapitulate human glioma morphologically, with a highly cellular tumor that has nuclear atypia present and stains strongly for GFAP.^20,82^

#### ***Pros***.

—The SMA-560 model has a few unique benefits that distinguish it from previous models discussed. In addition to recapitulating human gliomas morphologically, as previously mentioned, the model also has a similar response to conventional treatments, especially TMZ. Interestingly, when evaluated in vitro, SMA-560 is resistant to temozolomide with a high EC_50_ of >500 μM.^83^ Corresponding with the in vitro data, temozolomide treatment alone in SMA-560 intracranially implanted mice, provides only a modest survival benefit.^83^ SMA-560 cells are slightly more sensitive to radiotherapy with significant reductions in cell viability and density as radiation dosing is increased.^83^ Similarly, and in contrast to human GBM, *in vivo* radiotherapy of established intracranial tumors alone provides a more robust benefit, with some long-term survivors.^83^ In addition, SMA-560 tumor cells also secrete the immunosuppressive molecule, TGF-ß, making it a valuable tool for research into the effects of immunosuppression on GBM immunotherapies.^23^

#### ***Cons***.

—Despite potentially being useful to study TGF-ß mediated immune suppression in the GBM microenvironment, the SMA-560 model remains moderately immunogenic, in contrast to human GBM. This immunogenicity is in part due to the relatively high mutational load of SMA-560 tumors as they contain 2171 non-synonymous exome mutations.^62^ In addition, while MHC I expression is low in SMA-560 at baseline, it is upregulated in cells exposed to interferon-gamma, further contributing to the immunogenicity of the model.^20^ SMA-560 is also modestly used in the literature relative to other models, such as GL261, and, as a result, is not as well characterized.

#### ***Uses***.

—In one of the earlier studies to use SMA-560, Sampson et al., used the model to demonstrate that the secretion of IL-2, IL-4, and tumor necrosis factor alpha (TNF α) lead to increased survival of VM/Dk mice following intracranial injection of modified tumors.^20^ Interestingly, IL-3, IL-6, Interferon-gamma, CD80, and granulocyte-macrophage stimulating factor did not affect mouse survival when similarly tested.^20^ A more recent study by Miller et al. used a similar experimental model to demonstrate that secretion of soluble CD70, an activator of the costimulatory receptor CD27, leads to prolonged survival of VM/Dk mice.^21^ Another study utilizing the SMA-560 model assessed chimeric antigen receptor (CAR) T cells specific for EGFvIII, resulting in a cure of the tumors and resistance to rechallenge.^22^ Following injection with EGFRvIII-specific CAR T cells, mice were resistant to rechallenge with EGFRvIII-negative tumors, indicating generation of an immune response against other tumor antigens, a potentially model-specific response given the relative plethora of neoepitopes in SMA-560 relative to human GBM.

#### ***SB28***.

—In contrast to the previous models described, the SB28 mouse model is a newer model that is thought to better recapitulate the tumor immunogenicity and microenvironment of GBM.^12,27^ The SB28 model was generated using sleeping beauty transposons to insert constructs targeting p53, RAS, and PDGF pathways.^12,27,84^ To generate the model, sleeping beauty transposon flanked pT2/CAG-NRasV12 and pT2/shp53/mPDGF constructs were injected into the right ventricle of neonatal C57BL/6 mice.^27^ Seven weeks following glioma induction, the brain tissue was harvested and a clonal line stably expressing luciferase was selected.^27^ This resulted in a highly tumorigenic model with 100% of mice injected with 1600 cells developing a tumor and with a median survival of 29 days. Histological analysis of SB28 tumors reveals high cellularity with some invasion of the brain parenchyma.

#### ***Pros***.

—The SB28 model has low immunogenicity relative to GL261 and the other syngeneic mouse models. While, as previously discussed, the GL261 model has over 4900 non-synonymous somatic mutations on whole-exome sequencing, SB28 tumor cells have 108 mutations, resulting in a total of 11 potential predicted neoantigens.^12,62^ SB28 tumors also have a relatively modest amount of tumor-infiltrating T cells, with PD-1 expression on 50% of CD8+ T cells in the tumor microenvironment. It is also worth noting the low MHC I expression by SB28 tumors, further reducing their immunogenicity. Thus, the low mutational load and number of tumor-infiltrating T cells seen in SB28 more accurately represent the conditions found within a human GBM, potentially making it a robust model for preclinical therapy development experiments, especially those pertaining to immunotherapeutics. Also matching what is seen in human GBM, SB28 has a poor response to checkpoint inhibitors. In a study by Genoud et al., mice injected with SB28 tumors were subjected to combination checkpoint inhibitor therapy with anti-PD-1 and anti-CTLA4. This resulted in no significant beneficial effect in SB28 carrying mice. In contrast, mice injected with GL261 cells and subjected to the same treatment demonstrated significant survival benefits, with 50% of the mice displaying long-term survival.^12^ This demonstrates how the SB28 model recapitulates not only the tumor mutational load and microenvironment of human GBM, but also the response to immunotherapy. The SB28 model will undoubtedly continue to see use in future studies, especially those investigating immunotherapies, given its more accurate recapitulation of the human GBM tumor microenvironment relative to other syngeneic mouse models.

SB28 seems to respond similarly to human GBM when treated with conventional treatments as well. An early report by Garcia et al., highlighted the resistance of SB28 to radiotherapy as mice with intracranial tumors treated with radiotherapy alone had a modest survival benefit with an increase in median survival from 18 to 21 days.^85^ SB28 tumors are also poorly responsive to treatment with TMZ alone, which extended median survival to 23 days compared to 19 days in control mice.^86^ The minimal benefit of TMZ and radiotherapy in SB28 is similar to that seen in GBM, and indicates that SB28 may be a robust model.

#### ***Cons***.

—Although the SB28 model seems promising, as mentioned above, the primary downside to the model is that it is relatively new, and its use in the literature is limited. This downside should be mitigated as the model continues to be used over time. In addition, SB28 is a homogenous cell line, in contrast to the significant intratumoral heterogeneity seen in human GBM. As a result, SB28, like any similar cell line, may not accurately model the impact of intratumoral heterogeneity on treatment response or the development of treatment resistance. Nevertheless, additional studies using this model are needed to evaluate how it performs across different experiment types.

#### ***Uses***.

—As previously mentioned, the SB28 model is newer than other models described in this section with relatively limited use in the literature thus far. Garcia et al., demonstrated an increased response to radiotherapy when combined with the TGF-B inhibitor 1D11, to a median survival of 31 days compared to 18 days in control mice. A similar increase in median survival was seen when 6 Gy of radiation therapy was combined with anti-PD-L1 therapy, suggesting that preemptive radiation can enhance the response to biological agents.^85^ Interestingly, a synergistic effect with biological agents was noted when TMZ was used in the SB28 model. TMZ, in combination with HOE642 (a NHEA1 inhibitor) and anti PD-1 treatment, further increased survival to 33 days, significantly higher than any treatment alone, highlighting the benefit of combination therapy.^86^ SB28 has also demonstrated a response to combination therapy with celecoxib and anti-CD40 treatment, though with less than 20% of mice demonstrating a long-term response compared to ~40% of Quad-GL261 mice.^27^

### A Brief Note on Luciferase as a Reporter Gene

The ability to monitor tumor models using non-invasive methods in vivo is undeniability valuable and provides additional insight into tumor implantation rates, growth kinetics, and more. A common way to assess tumor size is through the use of cells that stably express firefly or Renilla luciferase, which produces light that can be measured through bioluminescent imaging when the luciferin substrate is given. Any non-murine protein used as a tumor marker, including luciferase, has the potential to act as a tumor antigen that the mouse immune system can target. If the presence of luciferase or another tumor tracker gene has any sort of effect on a tumor model is an area of interest within cancer immunology with no clear answer. Studies using the 4T1 breast cancer model have demonstrated that luciferase likely acts as an antigen, leading to the restriction of tumor cell growth and metastasis as a result of T cell activities.^87,88^ In contrast to these findings, a study using the ID8 model of ovarian cancer showed no effect of luciferase expression on tumor engraftment or the tumor immune microenvironment.^89^ Similarly, in the GBM GL261 model, Clark et al. showed no difference in proliferation, cytokine expression, invasive characteristics, CD3+ cell infiltration, or in vivo growth between tumor cells expressing luciferase and those without luciferase.^90^ However, a more recent study by Sanchez et al. highlighted improved survival in mice injected with GL261 tumors expressing luciferase with or without an additional fluorescent protein marker, relative to wild-type tumor cells.^91^ They also showed increased inflammatory immune cells in tumors expressing luciferase and a fluorescent protein marker as well as increased secretion of inflammatory cytokines by these cells.^91^ Given the mixed literature on the subject, attention should be paid to the use of luciferase or other nonendogenous tumor tracking proteins, such as GFP, especially if investigating immunotherapies. Imaging techniques like magnetic resonance imaging (MRI) or potentially the use of radioactive imaging using tumor cells expressing the murine sodium-iodine symporter (NIS) can provide non-immunogenic solutions of monitoring tumor growth, though they are limited by the cost and availability of the technologies.^92^

## Genetically Engineered Mouse Models

### Introduction to Genetically Engineered Mouse Models of GBM

GBM, like many other cancer types, is heavily influenced by genetic alterations and mutations.^93,94^ Genetically engineered mouse models (GEMMs) of GBM harness molecular biology techniques, such as tet and cre systems, to alter the genetics of mice and drive tumor formation.^44^ GEMMs can be created using a number of different methods, including conventional knockouts, conditional knockouts, transgenic mice, RCAS (Replication Competent ALV LTR with a Splice acceptor), and other viral techniques.^95^ A newer methodology to create GBM GEMMs also includes the utilization of clustered regularly interspaced short palindromic repeats (CRISPR) Cas9 technologies. This section will discuss the general characteristics as well as the advantages and disadvantages of GEMMs, as there are a variety of other substantial review articles containing more granular information regarding each individual model.^44,96^

### Genetically Engineered Mouse Models of GBM

The majority of GEMM research for GBM has focused on investigating the role of underlying genetic alterations in GBM tumorigenesis,^42,44,45^ but there are a number of studies using GEMMs as a model for the evaluation of therapeutics.^46–50^ Compared to other model types, GEMMs are more frequently used for the evaluation of GBM genetics or therapies that are specifically targeted against genetic mutations.

#### ***Pros***.

—GEMMs have significant advantages relative to some of the other models discussed in this review. Unlike xenograft models (discussed below), GEMMs allow GBM to be modeled in mice with an intact immune system, allowing for investigations into immunotherapies and the role of immune cells in tumor biology. GEMMs also more accurately recapitulate tumor formation as they do not involve the injection of tumor cells directly into the brain, avoiding disruption of the blood-brain barrier and the inflammation associated with tumor injection. Rather, they progress through tumor development in a manner more similar to human GBM.^30^ GEMMs also allow investigators to directly evaluate how mutations in specific genes (like PTEN or P53) can impact sensitivity to treatment.^48^ For example, Lin et al. used p53;p16^Ink4a^/p19^Arf^;K-Ras^v12^;LucR and a Pten;p16^Ink4a^/p19^Arf^;K-Ras^v12^;LucR (p53 or PTEN loss with concomitant p16 loss, p19 loss, and K-Ras expression) models to investigate the treatment response to a PARP inhibitor ABT-888 combined with TMZ. Interestingly, they found the combination treatment was more effective in the PTEN model than the p53 model, potentially highlighting a subgroup of patients that may benefit in the clinic.^48^ This study highlights the unique versatility of GEMMs and their utility in interrogating various aspects of GBM biology.

#### ***Cons***.

—There are, however, a number of disadvantages associated with GEMMs that can make some investigators use alternative methods, especially in the setting of testing novel therapeutics. One of the most significant disadvantages of GEMMs is that they can frequently lack the intratumoral heterogeneity seen within GBM, given their comparatively reductionist mechanism of formation.^28,30,44^ Traditional GEMMs can also require sophisticated breeding strategies and tumors can often be slow to form, making their use expensive and challenging at times, although some modern techniques have been able to reduce these shortcomings.^28^ In addition, tumors can develop in various locations within the mouse brain, in contrast to direct intracranial injections, limiting the ability to test some delivery modalities, such as convection-enhanced delivery (CED) which requires precise placement of a catheter in the tumor.^97^

#### ***Additional uses***.

—A variety of genetic perturbations can be explored using GEMMs. One of the earliest GBM GEMMs created through the loss of tumor suppressor genes included the loss of NF1 and TP53, leading to mice that developed a range of astrocytomas, from low to high grade.^98^ Subsequent models have utilized RCAS to investigate PDGF-B genetics- providing mice with oligodendrogliomas of varying grades.^99^ Similarly, GEMMs have been used to investigate the role of EGFRvIII and PTEN mutations, which are common in GBM, in GBM pathogenesis.^100^ Wei et al. demonstrated that both overexpression of EGFRvIII and PTEN inactivation in the RasB8 glioma-prone mouse strain, potentiated higher grade gliomas; this provided additional insight into their role in GBM and provided a model in which therapies targeted against EGFRvIII and PTEN could be tested, further highlighting the utility of GEMMs. Lineage tracing can also be utilized in GEMMs for a multitude of different investigations. Liu et al. utilized Mosaic Analysis with Double Markers (MADM) to explore the cell of origin in gliomagenesis. Lineage tracing following the generation of concurrent p53/Nf1 mutations sporadically in neural stem cells (NSCs) highlighted oligodendrocyte precursor cells (OPCs) as the cells of origin in their particular model, demonstrating the utility of these techniques.^101^

Novel technologies such as CRISPR/Cas9 have also increased the flexibility and granularity of GEMMS.^102^ CRISPR/Cas9 has been used to create a variety of GEMMS with single and multiple gene mutations.^103,104^ This is highlighted by Oldrini et al. who utilized CRISPR/Cas9 to knock out either a single tumor suppressor gene, PTCH1, or multiple genes TRP53, PTEN, NF1, to create models of medulloblastoma and GBM, respectively.^103^ Exciting applications of this technology include in vivo CRISPR/Cas9 screens. For example, Chow et al. utilized stereotactic intracranial injections of an adeno-associated virus-mediated genetic CRISPR screen to evaluate functional tumor suppressor genes in glioblastoma. While the low engraftment rates and number of cells injected per mouse during the establishment of many GBM cell lines limit the breadth of in vivo CRISPR screens that can be performed utilizing ex vivo genetic manipulation, viral delivery systems injected intracranially, such as the one utilized by Chow et al. are interesting candidates for in vivo screens in GBM.^105^

## Xenograft Models of GBM

### Introduction to Xenograft Models of GBM

Xenograft models of GBM seek to model human GBM via human GBM tumor cells that are implanted into immunodeficient mice, such as nude, NOD/SCID mice, and NOD/SCID gamma (NSG) mice. Xenograft models have traditionally been generated using human GBM cell lines that are maintained in culture then injected into mice; this includes the highly used U87 and U251 cell lines. Patient-derived xenografts are a technique involving the generation of a xenograft model directly from a patient GBM tumor sample that is viewed more favorably, given the increased ability to faithfully recapitulate human GBM in a mouse model.

### Human Xenograft Cell Lines

#### *Advantages and disadvantages of xenograft human glioblastoma cell lines*.

—Human GBM cell lines, including U251 and U87, have been used extensively in thousands of publications since their conception in the 1960s.^106,107^ Similarly, additional human GBM cell lines, such as LN229, LN18, and T98G have seen broad use in the literature.^108^ Human GBM cell lines are easy to work with, can be maintained for extended periods of time in cell culture, and mimic human GBM histopathology. Given their similarity to human GBM, relative to other model types, they are frequently used to investigate tumor-specific signaling pathways such as intracellular growth systems, apoptosis signaling, and angiogenesis, as well as treatments targeting these pathways and mechanisms of treatment resistance.^109^

Disadvantages of these models include their limited use in assessing immunotherapies, given their need to be modeled in immunodeficient mice. Xenograft cell lines are also comprised of homogenous cell populations, lacking the incredible intratumoral heterogeneity seen in patient GBM cases, thus limiting their ability to recapitulate a human GBM. Similar to other murine GBM models, human GBM cell lines face genetic drift and alterations in their transcriptomes following prolonged cell culture with serum, losing some of their ability to model human GBM accurately.^110^ This can be avoided by maintaining cells in serum-free neural stem cell media, allowing for the maintenance of a human GBM phenotype.^111^ Unfortunately, recent reports detailing differences in the DNA profile of widely distributed “classic” human glioblastoma cell lines and the original cells,^43^ have increased concerns regarding the standardization and reproducibility of some of these lines.

### U87

The U87 line is one of the most widely used human GBM cell lines in the literature and is used in over 2000 publications.^30^ It was initially established from a woman with GBM in the 1970s. While widely used and distributed, the U87 line does have some differences when compared to human GBM on histopathology, which may impact its response to treatments.

#### *Pros*.

—A benefit of the U87 line is their widespread use throughout the literature, as previously discussed. In addition, the U87 model has a population of CD133+ cells, allowing them to form neurospheres and be used in the study of glioblastoma tumor stem cells.^112^ Genetically, U87 also demonstrates some similarities to human GBM and carries hTERT and ATRX mutations, both of which can be seen in human GBM. U87 cells do not carry p53 or IDH1 mutations, but do have a mutation in PTEN and have a methylated MGMT status, which both can be found in human GBM as well.^40,41^

#### *Cons*.

—The U87 model has a large number of disadvantages. In contrast to human GBM, U87 tumors implanted into mice are well demarcated and surrounded by reactive astrocytes without diffuse infiltration.^32,33,42^ The lack of tumor infiltration is a significant limitation of the U87 line as diffuse infiltration is a key feature of human GBM. The U87 tumor vasculature also has higher levels of “leaky” vessels, relative to human GBM, potentially increasing the access of systemic drugs to the tumor microenvironment.^42^ In addition, U87 tumors have relatively rare necrotic features, without pseudo-palisading patterns or neutrophil infiltration.^35^

Also in contrast to human GBM, U87 cells respond to both radiation and TMZ treatments.^63,113–121^ In cell culture, U87 cells die in a dose-dependent manner to radiation therapy, with approximately 10% of cells remaining viable after 10 Gy of radiation.^120^ In vivo studies correspond with this finding, with radiotherapy similarly decreasing tumor burden and improving survival in subcutaneous and intracranial models.^119,121^ Similar to radiation therapy, U87 cells respond to TMZ treatment, though reported IC_50_ values vary in the literature from 7–204 uM.^114–117,122,123^

An additional disadvantage of the U87 line is concern surrounding its authenticity. In fact, the authenticity of widely available U87 cell lines from American Type Culture Collection (ATCC) was challenged when the lab that initially isolated the U87 cell line found that U87 cell line widely available from ATCC had a different DNA profile from that of the original cells.^43^ Subsequent analysis revealed that the U87 line from ATCC was of CNS origin and likely a GBM line, though of unclear origin. Thus, while the contributions from the U87 line to neuro-oncology are substantial, the field has shifted away from using the U87 line given concerns regarding authenticity, reproducibility, and due to key differences in histopathology between U87 and human GBM.

#### *Uses*.

—The U87 model has been used to study a variety of topics within GBM tumor biology. Anti-angiogenic therapies, including underlying molecular underpinnings of tumor responses and resistance as well as dosing, have frequently been tested using the U87 model.^37^ Pechman et al. used the U87 model to provide insight into bevacizumab dosing, demonstrating that, when compared to lower doses, only a maximum dose of 10 mg/kg resulted in decreased tumor growth.^37^ The U87 model has also been used to provide insight into the molecular response of tumor cells to bevacizumab. Using a U87 bevicizumab resistant model (BevR), Jahangiri et al. highlighted the importance of the c-met/B1 integrin complex in driving the increased invasive properties of BevR cells, thus potentially providing a new therapeutic target for the prevention of increased GBM invasion in response to bevacizumab.^124^ The U87 line has also been used to evaluate an extensive range of potential treatments from pharmaceuticals such as siroliumus and chloroquine in combination with temozolamide^38^ to neural stem cells carrying tumoricidal gene products.^39^

### U251

Similar to the U87 model, the U251 cell line was isolated in the 1970s from a 75-year-old male with GBM.^125^ The U251 model has been used extensively in both intracranial and subcutaneous models since its isolation and has been published in over 1000 studies.^30,31^ Following inoculation, the intracranial U251 model recapitulates the histopathology of human GBM well.^32–35^

#### *Pros*.

—U251 models have a number of advantages, including a strong recapitulation of human GBM histopathology. Unlike U87, U251 models display infiltrative invasion, palisading necrosis, cellular atypia, and mitotic figures, as well as edema and hemorrhage.^32,33,35^ However, unlike human GBM, the invasion seen does not occur along white matter tracts.^32^ U251 cells have high levels of proliferation, with the majority of cells staining for Ki-67.^33^ Like U87, a subset of U251 cells express CD133 and are able to form neurospheres, allowing for the propagation of glioblastoma tumor stem cells.^112^ Genetically, U251 cells do not carry an IDH1 mutation, but do carry hTERT, PTEN, and p53 mutations and have a methylated MGMT status, which can also be seen in human GBM.^41^

#### *Cons*.

—In contrast to human GBM, U251 is known to be responsive to both TMZ and radiation treatments.^126–133^ When irradiated in cell culture, U251 displays a dose-dependent response to radiation with a surviving fraction of less than 0.1 following a radiation dose of 6 Gy.^128^ In vivo experiments also highlight the responsiveness of the U251 model with 3 treatment episodes of 6 Gy in one week, providing a 20% survival benefit in mice with intracranial U251 tumors.^128^ U251 tumors are similarly responsive to TMZ, with IC_50_ values varying in the literature from <20 μM to <500 μM.^117,130–133^ A systematic review and meta-analysis by Hirst et al., demonstrates a survival ratio (treated survival divided by control survival) of approximately two for in vivo U251 models treated with TMZ.^134^

Unfortunately, in 1999, the U373 GBM line was reportedly cross-contaminated by U251 cells.^32^ Like U87, and considering the past cross-contamination, recent reports have questioned the authenticity and validity of widely available U251 cell lines. Indeed, long-term subclones of U251 accumulated genetic changes and experienced genetic drift resulting in a variety of phenotypic changes relative to the original U251 line.^36^ These changes include differences in cell morphology, cell surface marker expression, and increased growth in vitro and in vivo.^36^ As a result, investigations using the U251 line should be approached with caution and the line should be verified prior to use.

#### *Uses*.

—U251 has seen extensive use in the literature and has been used to study a variety of topics within GBM, including as a model to investigate new treatments and GBM physiology. This includes the study of alkylating agents, including temozolomide, lomustine, and carmustine as well as the anti-angiogenic small molecule drug cilengitide.^28^ U251 has also been used to study improvements on current therapies such as direct intracranial delivery of bevacizumab^135^ and the synergism between metformin and temozolomide^29^ as well as insights into GBM behavior.^136^ Similarly to U87, U251 will likely see a reduction in use given concerns over its ability to accurately recapitulate human GBM and reproducibility as well as the growth of improved models, such as PDX models.

### Patient-Derived Xenografts

The preclinical efficacy of a specific treatment remains a poor predictor of eventual clinical efficacy across many cancer types, including glioblastoma.^10^ A large part of this is assumed to be due to the inability of “traditional” preclinical models, such as the U87 model in GBM, to accurately recapitulate human GBM biology and heterogeneity in an animal model. A 2009 study by Daniel et al., demonstrates the loss of tumor-specific genes when small cell lung cancer biopsies are passaged in culture relative to the original patient biopsies and biopsies immediately implanted into mice. Tumor-specific genes were not regained when the cell lines generated were subsequently implanted into mice, highlighting the potential for long-term genetic and functional repercussions following exposure of patient tumor samples to standard cell culture techniques.^137^ Indeed, tissue culture conditions have similarly been shown to have a significant impact on glioblastoma tumor stem cells and their subsequent genetics and phenotypic outcomes. Lee et al. demonstrated the harm caused by standard cell culture techniques when applied to GBM. In their study, following harvest from a patient, tumor cells were processed into single-cell suspension, then grown in either traditional neural stem cell media (serum-free media containing bFGF and EGF) or DMEM with 10% FBS. Cells grown in serum-free media retained a similar genotype and phenotype to the primary tumor cells, while those grown in the presence of serum gained genetic alterations, eventually resulting in a cell line that was significantly different than the original tumor.^110^ This study highlights the importance of cell culture conditions and also demonstrates the potential utility of PDX models for accurately replicating human GBM in a mouse.

PDX models are those in which tumor samples are taken from a patient, processed into pieces or a single cell suspension and immediately injected into a mouse either in the ectopic subcutaneous microenvironment or in the orthotopic intracranial microenvironment.^57,138–141^ In the case of GBM, it is also acceptable to grow cells in serum-free media supplemented with bFGF and EGF then inject them into mice, given the genetic stability of GBM tumor cells grown in this manner.^57,110^ Of course, as they are derived directly from human cells, PDX models should be implanted into immunocompromised mice, including nude mice, NOD/SCID, and NSG mice to prevent tumor rejection. Databases of annotated PDX models, such as the NCI patient-derived models repository^142^ and the Charles River Tumor Model Compendium^143^ have become publicly available. Several groups have established and thoroughly characterized a significant proportion of the GBM PDX models currently available, including the Mayo Clinic, which established 94 PDX lines from 261 GBM patients undergoing surgery at Mayo Clinic between May 2000 and May 2017, naming the successfully established PDXs based on which case number they corresponded to, from GBM3 to GBM229.^144^

#### *PDX implantation location*.

—An important consideration when utilizing PDX based models is the location of tumor implantation. PDX models are also not invulnerable from the issues with genetic drift and human GBM recapitulation facing other GBM models. For instance, a 2017 study by Ben-David et al. that investigated dynamic copy number alterations (CNAs) across 1110 PDX samples from 24 cancer types, including GBM, found that some CNAs that were repeatedly observed in primary human tumors were selected against in PDX models within early passage numbers in mice. Some acquired CNA alterations were then demonstrated to impact the response to therapies, highlighting potential repercussions on preclinical therapeutic testing.^145^ However, this study was primarily performed using the subcutaneous implantation of PDX cells, which may exert unique microenvironmental pressures on tumor growth leading to divergent tumor evolution away from the genetics of primary human tumors. A subsequent study by Golebiewska et al. examined the genetic, epigenetic, transcriptomic, and histopathological stability of patient-derived orthotopic xenografts (PDOXs) that were briefly grown in culture and allowed to form organoids following initial collection, then intracranially implanted into mice.^146^ In contrast to Ben-David et al., they demonstrated the long-term stability of GBM PDOXs with a consistent recapitulation of primary tumor characteristics and no evidence of mouse-specific tumor evolution.^146^ PDOXs also had clinically relevant responses to medical therapies such as TMZ, further highlighting the importance and utility of PDOXs.^146^ It is also important to note that Golebiewska et al. only cultured patient-derived cells for two weeks prior to implantation, then continued PDOX model generations with minced xenograft brains that were directly implanted into the next generation of mice, therefore limiting any potential tumor evolution to the cell culture environment. Given their accurate representation of primary human tumors, PDOXs may be used as avatars of human tumors to measure and predict therapeutic responses to novel treatments.

#### *Pros*.

—The most significant advantage of GBM PDX models is their faithful recapitulation of human GBM features. In a 2009 study, Wakimoto et al. demonstrated that, like human GBM, PDX models result in tumors that invade the brain following orthotopic injection into mice.^147^ Additional studies using GBM PDX models have also demonstrated the recapitulation of characteristic human GBM findings such as endovascular proliferation, pseudopalisading necrosis, and diffuse invasion through the brain with some models demonstrating extension through the corpus callosum and into the contralateral brain.^51,56^ PDX models even demonstrate subtype-specific characteristics and growth patterns. PDX models generated from cells of the mesenchymal subtype (MES) proliferate at a higher rate following implantation and exhibit increased vascularity and invasiveness relative to those generated from cells of a proneural (PN) subtype, mirroring human GBM physiology.^56,141,148,149^

#### *Cons*.

—Though currently experiencing substantial use and considered one of the most optimal models for human GBM, PDX models have certain disadvantages. While a plethora of protocols exist in the literature, establishing a PDX line is not always successful and is dependent on the experience of the lab.^57,58^ Also, as each PDX model is derived from a different patient sample, there can be significant variability between different individual models, limiting their standardization and potentially reducing the reproducibility of experimental results.^58^ Another significant disadvantage is the need to use PDX models in immunodeficient mice, making it impossible to investigate the role of the immune system in tumor physiology or treatment response without humanized mice. Finally, as previously discussed, subcutaneous implantation of PDX models can lead to mouse-specific tumor evolution and poor recapitulation of primary tumor characteristics.^145^

#### *Uses*.

—As a result of the advantages associated with PDX models, they have been used to study a variety of topics within GBM, such as tumor biology and the therapeutic potential of new treatments.^51,53,56,147,150^ PDX models have shed light on the role of glioma stem cells in human brain tumors and how they contribute to radioresistance in GBM.^51,52^ They have also been used to investigate a variety of potential drugs including bevacizumab, temozolomide, and veliparib as well as underlying molecular mechanisms behind sensitivity and resistance.^53–55^ In addition, PDX models have seen use in evaluating oncolytic herpes simplex virus vectors as well as high throughput screens for new drug treatments.^147,150^

## Future Directions

Preclinical models that accurately recapitulate human GBM are key in developing new therapeutics with a chance for clinical success. While current murine models have aided in new therapy development, they each have disadvantages that limit their clinical translatability. As the field moves towards more combination treatments, the ability to test and quickly identify optimal combinations will become increasingly critical. Inherent disadvantages of murine models include the large amounts of time and resources needed to evaluate novel therapeutics. New models such as cerebral organoid models of GBM,^151^ organotypic slice cultures of GBM,^152^ or computational models^153^ may have a prominent role in future investigations and should be carefully considered; they may allow for cheaper and faster interrogations of various therapies and combinations. However, for now, these models lack the complexity of murine systems, especially with regards to recapitulating the GBM/immune interface and allowing for the accurate evaluation of immunotherapies. Thus, novel modeling systems will likely continue to see increased usage in evaluating chemotherapies and other treatments that don’t garner the majority of their treatment effect from the immune system. However, immunotherapies will continue to require murine models for evaluation of their efficacy until the GBM/immune interface can be faithfully reproduced in silico or in vitro. Murine models also continue to improve and move towards better replicating the conditions of a human GBM tumor. In fact, a newer development within cancer immunology, and neuro-oncology, is the use of PDXs established in humanized mice with a human immune system.^154^

As previously discussed, PDXs can be excellent models in that they can most accurately replicate human GBM with regards to histological findings and intratumoral heterogeneity of the cancer cells. A significant disadvantage of current PDX models is the need to be implanted in an immunodeficient mouse. It is well known that the immune system can play an essential role in GBM response to treatment and many treatments, such as immunotherapies, rely on the immune system to play an active role in tumor killing.^155^ Thus, the lack of an immune system in PDX models is a severe limitation. Fortunately, PDX models can be implanted in humanized mouse models, immunodeficient mice with a human immune system, are gaining popularity in cancer immunology.^154^ These models can also be generated with peripheral blood mononuclear cells (PBMCs) derived from the same patient as the GBM PDX, recapitulating that patient’s immune-GBM interface.^154^ A study by Ashizawa et al. used a humanized mouse model to investigate the effect of Programmed Death-1 (PD-1) blockade on intracranial U87 tumor rejection, demonstrating a reduction of 50% in tumor size and highlighting the potential uses of humanized mouse models.^156^ Studies involving GBM PDX models in humanized mice will likely play a role in future preclinical studies, especially those evaluating immunotherapies.

## Conclusion

GBM remains incurable with a median survival of 12 to 15 months^157^ and a standard of care that has remained unchanged for over a decade. Preclinical mouse models of GBM, including syngeneic models, GEMMs, and human xenograft models have a crucial role in learning more about GBM biology as well as developing and testing novel therapies. Each model type has advantages and disadvantages that should be considered when designing and interpreting preclinical studies (Table 2). Table 2 summarizes the unique characteristics of the cell lines discussed in this review.

**Table 2.** Summary of GBM Cell-line Models

Cell Line	Mouse Strain	Average Survival	Mutational Load	MHC I expression	XRT sensitivity	TMZ sensitivity	Genetics
Human GBM		15 months with treatment	- Low^7–9^	- Low-^7–9^	- Low	- Low	- Most common mutations include TP53, PTEN, EGFR^158^
GL261	C57BL/6	31 days following injection with 5 x 10^4^ cells	- High- over 4,932 non-synonymous exome mutations - High number of predicted neoepitopes^62^	- High^62^	- 2 Gy needed for 50% cell death in vitro.^19^ - 4 Gy in brain tumor bearing mice slows growth without cure.^19^	- EC_50_ of 400 μM in vitro.^64^ - Prolongs survival without long term survivors in vivo.^65^	- Elevated p53 expression, carry a p53 point mutation^19,68^ - Mutation in the K-Ras oncogene and elevated c-myc expression^19^ - Wild-type cytosolic isocitrate dehydrogenase (IDH1)^69^
SMA-560	VM/Dk	25 days following injection with 5 x 10^4^ tumor cells	- High- 2,171 non-synonymous exome mutations^62^	- Low-Upregulated in response to IFN-gamma^20^	- Sensitive to radiotherapy in vitro. - XRT alone can lead to long-term survival in vivo.^83^	- Resistant to TMZ in vitro. EC_50_ of >500 μmol/L. ^83^	- Limited research
CT-2A	C57BL/6	20 days following an intracranial injection of 1 x 10^4^ cells has been reported as 20 days	- Poorly described - Likely high given chemical induction generation	- Expresses MHC I with upregulation in response to IFN-gamma^78^	- Poorly reported in the literature though frequently describe as chemo and radioresistant.^74,79^	- Poorly reported in the literature though frequently describe as chemo and radioresistant.^74,79^	- p53 wild-type and PTEN deficient.^74–76^
SB28	C57BL/6	23 days following injection of 5 x 10^3^ cells	- Low- 108 mutations, resulting in a total of 11 potential predicted neoantigens.^12,62^	- Low MHC I expression^12,62^	- Modestly responsive to XRT—10 Gy of radiation provides an increase in median survival from 18 to 21 days in vivo.^85^	- Moderately responsive to TMZ—TMZ alone extends median survival from 19 to 23 days in vivo.^86^	- Generated using sleeping beauty transposons to insert constructs targeting p53, RAS, and PDGF pathways.^12,27,84^
U251	Nude/NOD SCID/NSG	22 days following injection of 1.5 x 10^6^ cells	- Unclear, high variability between cell lines, length in cell culture, questions regarding authenticity^36^	- Low MHC I expression.^159^	- Dose-dependent response to radiotherapy with a surviving fraction of less than 0.1 following a radiation dose of 6 Gy in vitro.^128^ - Moderately responsive in vivo—3 treatment episodes of 6 Gy in one week, provide a 20% survival benefit in mice with intracranial U251 tumors.^128^	- Significant variability in IC_50_ values varying in the literature from <20 μM to <500 μM.^117,130–133^ - Responsive to TMZ in vivo—survival ratio of 2 for in vivo U251 models treated with TMZ.^134^	- hTERT, PTEN, and p53 mutations - Methylated MGMT status - wtIDH1
U87	Nude/NOD SCID/NSG	28 days following injection of 1.0 x 10^6^ cells	- Unclear, high variability between cell lines, length in cell culture, questions regarding authenticity^43^	- Moderate MHC I expression.^159^	- Dose-dependent response to radiotherapy in vitro, with approximately 10% of cells remaining viable after 10 Gy of radiation.^120^ - Intracranial radiotherapy of U87 tumors slows tumor growth and increase survival.^119,121^	- Responsive to TMZ treatment. Reported IC_50_ values vary from 7–204 uM.^114–117,122,123^	- PTEN mutation - methylated MGMT status.^40,41^

Accurate recapitulation of human GBM is vital to increase the clinical translatability of preclinical studies. The lack of such recapitulation is evident in the number of successful preclinical treatments that subsequently failed in clinical trials. As biological techniques continue to advance, newer models such as PDX GBM models in humanized mice, as well as others, will potentially play a more significant role in preclinical studies. Given the range of new potential therapies to treat GBM, mouse models will continue to be an essential tool that researchers can use to evaluate their efficacy and safety.
